# Short-term efficacy and safety of recombinant human adenovirus type 5 combined with PD-1 immune checkpoint inhibitors and SOX regimen in neoadjuvant therapy of locally advanced gastric cancer: a retrospective study

**DOI:** 10.3389/fonc.2026.1851169

**Published:** 2026-07-15

**Authors:** Xue Ding, Zhengye Wang, Hong Zhang, Li Dai, Huaxing Ma, Guangxian Leng, Yunshan Yang, Yongjin Zhou, Siqi Luo, Qian Wang, Xiangren Jin

**Affiliations:** 1 Department of Gastrointestinal Surgery, Affiliated Hospital of Guizhou Medical University, Guiyang, Guizhou, China; 2 Guizhou Medical University, Guiyang, Guizhou, China; 3 Guizhou Provincial Key Laboratory for Digestive System, Guiyang, Guizhou, China; 4 Department of Hepatobiliary Surgery, The Affiliated Hospital of Guizhou Medical University, Guiyang, Guizhou, China

**Keywords:** gastric cancer, interventional perfusion, neoadjuvant therapy, oncolytic virus, recombinant human adenovirus type 5 (H101)

## Abstract

**Background:**

To evaluate the short-term efficacy and safety of recombinant human adenovirus type 5 (H101) combined with S-1 plus oxaliplatin, PD-1 immune checkpoint inhibitors(SOXP regimen) in the neoadjuvant treatment of patients with locally advanced gastric cancer (LAGC). Evaluate the safety of H101 in the treatment of gastric cancer via interventional perfusion administration.

**Methods:**

A retrospective study was conducted, enrolling patients with LAGC who received H10 combined with the SOXP regimen at The Affiliated Hospital of Guizhou Medical University between January 1, 2025, and August 30, 2025. During the first cycle of neoadjuvant therapy, H101 was administered via interventional perfusion chemotherapy targeting the gastric cancer. After patients completed two cycles of neoadjuvant treatment, tumor downstaging was assessed, followed by surgical intervention. The primary endpoint of the study was the postoperative pathological response rate (pCR); secondary endpoints included the objective response rate (ORR), duration of response (DOR), and the incidence of adverse reactions.

**Results:**

A total of 25 patients were included in the analysis. Among them, 8 patients (32%) achieved a complete response (CR), 16 patients (64%) achieved a partial response (PR), and 1 patient (4%) achieved stable disease (SD). The objective response rate (ORR) and disease control rate (DCR) were 96% and 100%, respectively, with an R0 resection rate of 100%. In terms of pathological response evaluation by tumor regression grade (TRG), 10 patients (40%) achieved grade 3, 14 patients (56%) achieved grade 2, and 1 patient (4%) achieved grade 1. The pathological complete response (pCR) rate was 40%. Most adverse events were mild to moderate and manageable, and no grade 4 adverse events were observed.

**Conclusions:**

This retrospective study indicates that the combination of H101 with the SOXP regimen exhibits promising short-term efficacy in the neoadjuvant treatment of LAGC, characterized by high ORR, DCR, R0 resection rate, and pCR rate. The preliminary evaluation shows that H101 administered via interventional therapy is safe in the treatment of LAGC. Additionally, the treatment-related toxicities are well-tolerated, supporting the potential clinical value of this therapeutic strategy for LAGC patients.

## 1 Introduction

China is a country with a high incidence of gastric cancer(GC), and its incidence and mortality rates both rank among the top globally. Approximately 40% of new gastric cancer cases worldwide occur in China annually (1).

Systemic chemotherapy has historically represented the cornerstone of treatment for advanced and locally advanced GC. Conventional regimens based on platinum compounds and fluoropyrimidines, such as the FOLFOX or XELOX (CapeOX) regimens, achieve overall response rates (ORR) of approximately 30%–50% in the first-line setting for advanced GC, with median progression-free survival (PFS) of 5–7 months and median overall survival (OS) of approximately 10–12 months (2, 3). In the perioperative and neoadjuvant settings, the FLOT (5-fluorouracil, leucovorin, oxaliplatin, and docetaxel) regimen demonstrated superiority over ECF/ECX in the FLOT4 trial, yielding a pCR rate of only 16% and a 3-year OS of 57% (4). Similarly, the SOX regimen (S-1 plus oxaliplatin), widely adopted in Asia, achieves neoadjuvant pCR rates of approximately 10%–20% (5, 6). These modest outcomes highlight the urgent need to explore novel strategies to enhance response rates and improve survival in LAGC.

Historically, the primary treatment for gastric cancer was immediate surgery, with an emphasis on extensive tumor resection and lymph node dissection. Currently, there is a predominant shift toward a surgery-centered multimodal treatment paradigm. Prior to surgery, targeted therapy, immunotherapy, and other modalities are employed to achieve tumor downstaging, followed by surgical intervention (5, 7–12).

Undeniably, the current upsurge in the development of immunotherapeutic agents has significantly improved the treatment of advanced gastric cancer compared with 10 years ago. In previous clinical studies showed immunotherapy combined with targeted therapy and chemotherapy has achieved certain efficacy in the treatment of advanced and metastatic GC (5, 7–12). However, some patients still have poor treatment outcomes. Many available targeted drugs show limited efficacy and cannot maintain therapeutic effects for a long time, which is attributed to the complicated tumor microenvironment (TME) and genetic instability (13). Immunotherapy requires selection of appropriate populations based on specific molecular markers; moreover, some GC patients achieve good clinical efficacy after initial immunotherapy but may experience recurrence (14). With the continuous development of clinical medicine and technology, surgical techniques have become increasingly mature. Therefore, the focus of subsequent treatment efforts is how to benefit more patients. Currently, how to convert the immunosuppressive TME of GC from a “cold” to a “hot” state to improve the efficacy of drug therapy and achieve tumor downstaging has become a key focus and research hotspot for clinicians.

Oncolytic viruses(OVs) can selectively infect and lyse tumor cells while releasing tumor-associated antigens (TAAs) to activate the host immune system, representing a promising strategy for converting immunologically “cold” tumors to “hot” tumors (15). For instance, specific oncolytic viruses disrupt the cell membranes of tumor cells, thereby facilitating the release of intracellular antigenic substances. These antigens are subsequently captured and processed by antigen-presenting cells (APCs); in turn, APCs activate T cells and potentiate the host’s immune attack against tumor cells (15).​

The combination of OVs with other immunotherapeutic agents (e.g., PD-1 immune checkpoint inhibitors) may exert a synergistic effect, enabling more effective reprogramming of the tumor immune microenvironment (TME) (15). Additionally, combinations of OVs with chemotherapy or targeted therapy can regulate the TME through distinct mechanisms, enhance immunotherapeutic efficacy, and further promote the conversion of “cold” tumors to “hot” tumors (15, 16). Existing studies have confirmed that various oncolytic viruses can transform the “cold tumor” state into “hot tumors” in multiple solid tumors, including liver cancer, head and neck tumors, and melanoma (17–20).

To date, several studies have confirmed this finding; however, research on OVs in GC remains scarce. In 2021, Professor Jiang’s team conducted a study comparing intratumoral injection of oncolytic virus combined with chemotherapy versus chemotherapy alone in patients with advanced GC, yielding some encouraging results (21). Currently, there are no reported studies on the combination of OVs with immunotherapy or targeted therapy in the field of GC.

Our center has previously conducted preliminary explorations into neoadjuvant therapy for LAGC. A prior study on immunotherapy combined with targeted therapy and chemotherapy for LAGC demonstrated that the pCR rate could reach nearly 22% (22). Nevertheless, a subset of patients failed to derive benefits from this neoadjuvant therapy. For instance, the findings observed in the Dargen IV study and another research conducted in Hebei Province indicated that a large number of patients still required more effective therapeutic interventions to achieve tumor downstaging, attain R0 resection, and ultimately improve their overall survival (6, 22). These limitations highlight the urgent need for novel therapeutic strategies for LAGC. OV therapy is poised to become one of the promising therapeutic approaches for cancer, as OVs offer the advantageous combination of tumor-specific cell lysis and immune stimulation to eliminate cancer cells, while sparing nonmalignant cells. We have previously conducted interventional chemotherapy for LAGC with certain positive outcomes. To date, the clinical application of OVs in gastric cancer has been restricted to local endoscopic injection. In this context, we aim to explore the feasibility of delivering OVs to LAGC via interventional approaches, with the goal of evaluating their safety and efficacy. Therefore, we retrospectively collected clinical data from 25 patients to summarize the short-term efficacy and the safety of OVs combined SOXP regimen.​ This study was approved by the Ethics Committee of The Affiliated Hospital of Guizhou Medical University.

## 2 Methods/experimental

### 2.1 Clinical data of patients

The clinicopathological data of 25 patients who received the combined regimen of recombinant human adenovirus type 5 (H101) and SOXP (S-1, oxaliplatin, PD-1 inhibitor) between January 2025 and August 2025 were analyzed. All patients provided signed informed consent before participation.

#### 2.1.1 Inclusion criteria

1.Aged 18–80 years, with pathologically confirmed gastric cancer (GC);​2.Tumors with a programmed death ligand 1 (PD-L1) combined positive score (CPS) ≥ 1;​3.No prior history of surgery, radiotherapy, chemotherapy, immunotherapy for GC;​4.No distant metastasis (M0 stage);​5.Clinical staging of cT3–cT4 and cN1–cN3 (per the latest TNM staging system for GC);​6.No underlying diseases (e.g., hypertension, diabetes mellitus, heart disease, thyroid disease);​7.Eastern Cooperative Oncology Group (ECOG) performance status score < 2;​8.Provided signed informed consent prior to study enrollment and met all inclusion/exclusion criteria.

#### 2.1.2 Exclusion criteria

1.Pregnant or lactating women;​2.Patients with severe mental illness;​3.History of other malignant tumors (except cured non-melanoma skin cancer or *in-situ* carcinoma);​4.Presence of acute infectious diseases, immune system-related diseases, or uncontrollable systemic diseases;​5.Known allergy to any component of the study treatment regimen.​

### 2.2 Neoadjuvant treatment

All patients received two cycles of neoadjuvant therapy prior to surgery (5).

First cycle: on day 1, recombinant human adenovirus type 5 (H101, 1.5×10¹² vp) and oxaliplatin (100 mg/m²) were administered via gastric arterial infusion (interventional perfusion). Following arterial infusion, patients received an intravenous infusion of a PD-1 immune checkpoint inhibitor over 30–60 minutes as a single dose on the same day. Tislelizumab was administered at a flat dose of 200 mg regardless of body weight. Sintilimab was dosed according to body weight as per its approved prescribing information for gastric and gastroesophageal junction adenocarcinoma: patients weighing <60 kg received 3 mg/kg, while those weighing ≥60 kg received a flat dose of 200 mg. From day 1 to day 14, S-1 was orally administered twice daily, with the dose determined by body surface area: 40 mg twice daily for BSA <1.25 m², 50 mg twice daily for BSA 1.25–1.50 m², and 60 mg twice daily for BSA >1.50 m².

Second cycle: on day 1, the same PD-1 inhibitor was administered intravenously over 30–60 minutes, using the identical dosing scheme described above (tislelizumab 200 mg flat dose; sintilimab 3 mg/kg for body weight <60 kg or 200 mg for body weight ≥60 kg). On day 2, oxaliplatin (130 mg/m²) was given as a 2-hour intravenous infusion. S-1 was administered orally from day 1 to day 14, using the same dose schedule as in the first cycle.

**Figure 1:**
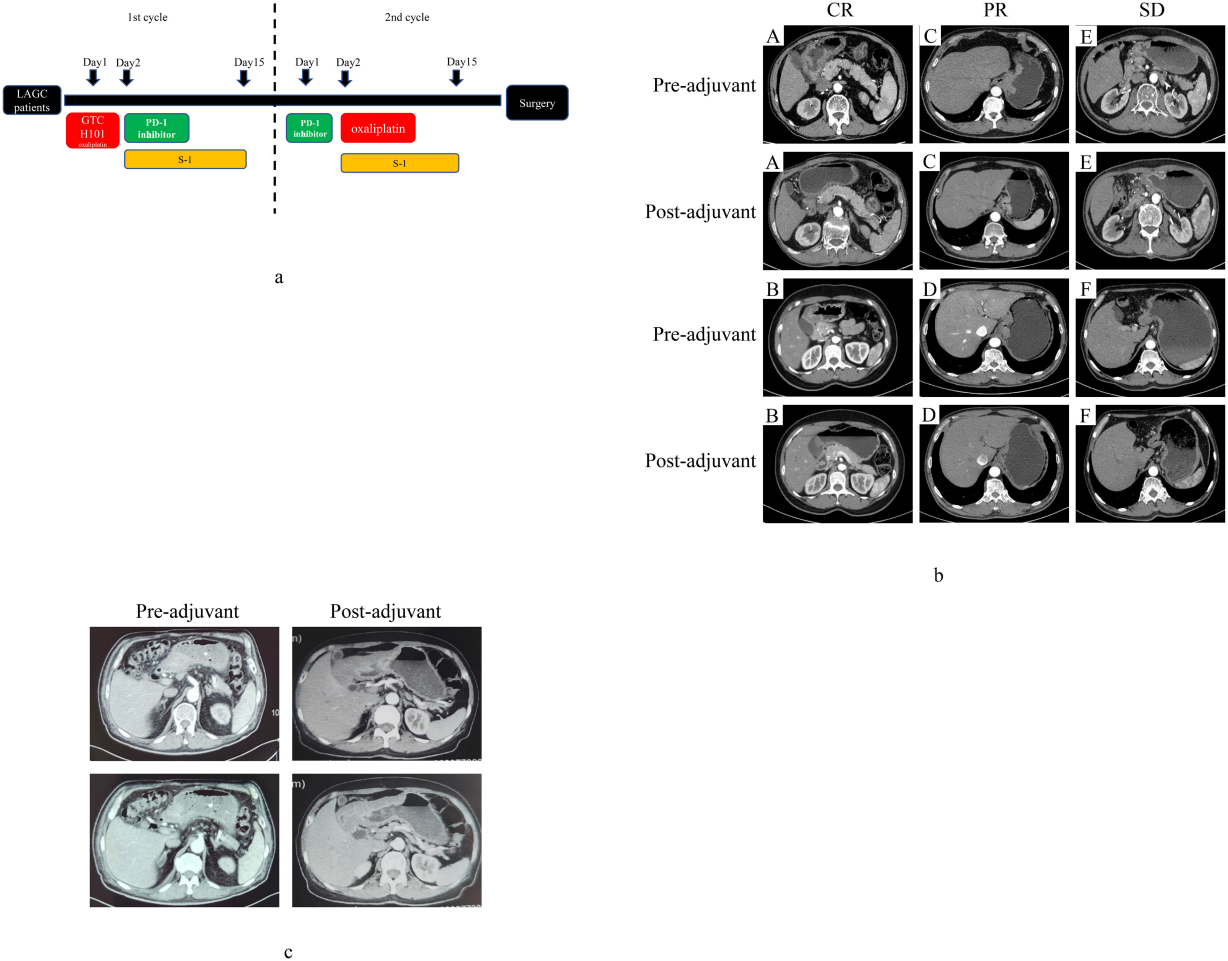
Neoadjuvant treatment protocol and radiological tumor responses in LAGC patients. **(a)** Neoadjuvant treatment protocol for locally advanced gastric cancer patients. **(b)** Abdominal CT scans depicting pre- and post-adjuvant tumor status across three response categories: complete response (CR, patients **(A, B)**), partial response (PR, patients **(C, D)**, and stable disease (SD, patients **(E, F)**). **(c)** Contrast-enhanced axial CT images from two representative LAGC patients showing pre- and post-adjuvant treatment comparisons. The upper panel illustrates a case with significant tumor regression following the two-cycle neoadjuvant protocol, evidenced by marked reduction in soft tissue mass and improved delineation of surrounding structures post-treatment. The lower panel presents a second case demonstrating notable morphological changes in the gastric region after neoadjuvant therapy, including decreased tumor burden and altered enhancement pattern.

The treatment process is shown in **Figure 1**.

### 2.3 Gastric transcatheter chemoembolization

GTC was conducted by experienced gastrointestinal surgeons. H101(1.5×10^12^vp) and oxaliplatin (100mg/m2) was used as an arterial chemotherapy and lipiodol (10mL) mixed with oxaliplatin (2mL) and H101(1.5mL) was used for embolism (21, 23, 24).

### 2.4 Endpoints and assessments

Preoperative efficacy was evaluated after 2 to 3 treatment cycles. In accordance with RECIST 1.1 (25), ORR is the proportion of patients with CR and PR, while DCR is the proportion of patients with CR, PR, and stable disease (SD).

After 2 treatment cycles, patients were assessed by the Multi-Disciplinary Treatment (MDT) team. Surgery was scheduled if the tumor achieved downstaging or if the patient opted for surgery after 2 treatment cycles.

All surgical procedures were performed by the same chief surgeon specializing in gastric surgery. A total of 25 patients underwent laparoscopic radical gastrectomy (LRG) for GC. Pathological responses in surgically treated patients were evaluated with reference to the Japanese Classification of Gastric Carcinoma, graded from 0 to 3 (26). The primary outcomes included pCR and the safety of perioperative treatment. Adverse events associated with the combination regimen were monitored and graded 0–4 based on the CTCAE version 5.0. The secondary outcomes were the R0 resection rate, ORR, and DCR.

SPSS 24.0 statistical software was used for analysis. Descriptive statistics were presented as the mean ± standard deviation. Oneway analysis of variance (ANOVA) was employed to determine significant differences among multiple groups, while the Chisquare test was employed to assess differences between the 2 groups. *P* <.05 was considered to indicate a statistically significant difference.

### 2.5 Assessment of CD8^+^ T cells

Blood samples were collected from each patient at two time points: at baseline (before the first cycle of neoadjuvant therapy) and on day 7 after H101 administration. CD8^+^ T cell absolute counts and percentages were determined by flow cytometry using the Tongsheng Shidai CD8^+^ T cell enumeration kit (Tongsheng Shidai Biotechnology Co., Ltd., China) according to the manufacturer’s instructions.

## 3 Results

A total of 25 patients with LAGC were enrolled, with a mean age of 65 ± 4.7 years. Among them, 15 (60%) were female and 10 (40%) were male (**Table 1**). Fourteen (56%) patients had Helicobacter pylori infection, and all patients (100.0%) were classified as clinical stage cT3 to cT4. Additional detailed baseline characteristics are presented in **Table 1**.

**Table 1:** Clinical characteristics of patients.

Clinical characteristics	Total (n=25)
Age(Year)	65 ± 4.7
Gender(n)	
Male	10
Female	15
Primary tumor location	
Fundus	5
Body	2
Pylorus	18
Pathological type(n)	
Adenocarcinoma	20
Signet-ring cell carcinoma	5
Depth of invasion before neoadjuvant treatment	
T3	15
T4a	10
Depth of invasion after neoadjuvant treatment	
T1	20
T2	4
T3	1
T4a	0
Lymph node metastasis before neoadjuvant treatment	
N1	3
N2	18
N3	4
Lymph node metastasis after neoadjuvant treatment	
N0	20
N1	5
N2	0
N3	0
Helicobacter pylori infection	14

### 3.1 Efficacy

**Figure 2:**
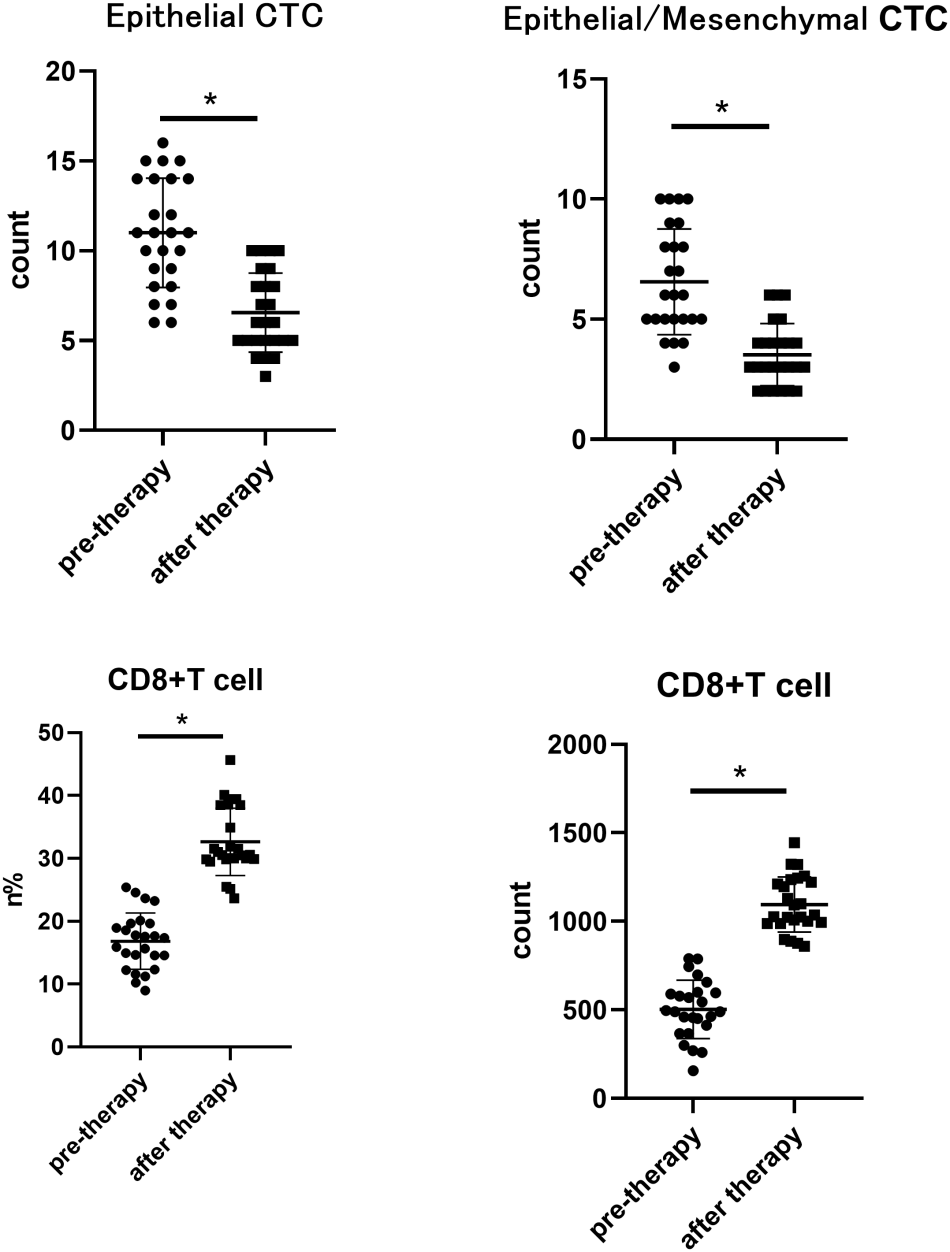
Changes in CD8^+^ T cells and circulating tumor cells (CTCs) before and after H101 administration. Absolute count and percentage of CD8^+^ T cells significantly increased following H101 administration compared with pre-treatment levels. Total number of epithelial CTCs and epithelial/mesenchymal CTCs significantly decreased after treatment. * indicates statistically significant difference (p < 0.05).

After efficacy assessment, 8 patients (32%)achieved CR, 16 patients (64%)achieved PR, 1 patients (4%) achieved SD, and no patient had PD (**Table 2**). The ORR rate was 96% (24/25) and the DCR rate was 100% (**Table 2**). Compared with the clinical stage before neoadjuvant therapy, 24 (96%) patients achieved T downstaging (**Table 2**). Furthermore, 96% of patients showed tumor regression, and all 25 patients (100%) achieved N downstaging, confirming the efficacy of the neoadjuvant therapy. Ten patients were downstaged from cT3 to cT1. Abdominal computed tomography images of representative patients in the CR, PR, and SD groups are shown in **Table 2**. Following H101 administration, the absolute count and percentage of CD8+ T cells increased significantly. In circulating tumor cell (CTC) detection, the total number of epithelial CTCs and Epithelial/Mesenchymal CTCs decreased significantly after treatment, No mesenchymal CTCs were detected in any of the patients (**Figure 2**).

**Table 2:** Clinical response of neoadjuvant chemotherapy.

Response	N (%)
CR	8(32%)
PR	16(64%)
SD	1(4%)
PD	0
ORR	24(96%)
DCR	25(100%)

We further compared the tumor regression outcomes between patients receiving sintilimab (n=14) versus tislelizumab (n=11). As shown in **Supplementary Figure 1**, tumor regression (defined as TRG 2–3) was achieved in 13 of 14 patients (92.9%) in the sintilimab group and in all 11 patients (100%) in the tislelizumab group, with no statistically significant difference (P = 0.87). This suggests that both PD-1 inhibitors are similarly effective when combined with the H101 and SOXP regimen.

### 3.2 Adverse events

Adverse events (AEs) in patients receiving neoadjuvant therapy were predominantly mild and clinically manageable (**Table 3**). All 25 enrolled patients completed the planned 2 treatment cycles without premature discontinuation. Common AEs included hematologic toxicities (leukopenia, anemia, thrombocytopenia), gastrointestinal reactions, elevated aminotransferase levels, fatigue, oral mucositis, hypertension, and limb numbness. Approximately 70% of patients experienced hematologic AEs, and a similar proportion (nearly 70%) reported gastrointestinal reactions.

**Table 3:** Adverse events during the neoadjuvant chemotherapy.

The type of Adverse events N(%)	Classification			Total
	1	2	3	
Fever	20(80%)	1(4%)	0	21(84%)
Hematologic Adverse Events				
Leukopenia	10(40%)	5(20%)	1(4%)	16(64%)
Anemia	7(28%)	5(20%)	1(4%)	13(34%)
Thrombocytopenia	4(16%)	5(20%)	0	9(36%)
Non-hematologic Adverse Events				
Nausea	11(44%)	5(20%)	1(4%)	17(68%)
Vomiting	5(20%)	6(24%)	1(4%)	12(48%)
Elevated transaminase	7(28%)	5(20%)	0	12(48%)
Abdominal pain	15(60%)	0	0	15(60%)
Oral ulcer	10(40%)	6(24%)	0	16(64%)
Hypertension	7(28%)	0	0	7(28%)
Proteinuria	0	0	0	0
Numbness	4(16%)	0	0	4(16%)
Nose bleeding	1(5%)	0	0	1(5%)

Notably, all AEs resolved completely following symptomatic treatment, with no Grade 4 AEs observed throughout the treatment period. Fever, a common AE associated with H101 administration, occurred in 21 patients (84%). Only 1 patient developed a fever exceeding 38.5°C, which responded promptly to ibuprofen and resolved uneventfully.

### 3.3 Surgical outcomes and postoperative pathological response

A total of 25 patients underwent surgery, all of which included D2 lymphadenectomy as part of the radical procedure. All patients achieved R0 resection and no 30-day postoperative readmissions, reoperations, or deaths were recorded. Pathological responses to neoadjuvant therapy were assessed according to the Japanese Classification of Gastric Carcinoma and graded from 0 to 3. Following neoadjuvant therapy, 10 patients (40%) were classified as Grade 3 (pCR), 14 patients (56%) as Grade 2, 1 patient (4%) as Grade 1, and no patients as Grade 0 (**Table 4**).

**Table 4:** Rates of surgical resection, R0 resection, pathological response.

Items	Patients (n=25)
Surgical resection No(%)	25(100%)
R0 resection No(%)	25(100%)
Pathological response (TRG grade, No (%)	
Grade0	0(0%)
Grade1	1(4%)
Grade2	14(56%)
Grade3	10(40%)
pCR No (%)	10(40%)

## 4 Discussion

To our knowledge, this is the first study to explore the combination of OVs with targeted therapy, immunotherapy, and chemotherapy for the neoadjuvant treatment of LAGC. When combined with the interventional administration route of OVs, the therapeutic efficacy was remarkably confirmed: 96% of patients achieved tumor downstaging, with an R0 resection rate of 100%. Specifically, 40% of patients attained pathological complete response (pCR), and the remaining 56% achieved partial response (PR), leading to a disease control rate (DCR) of 100% and an objective response rate (ORR) of 96%.

Notably, these outcomes represent a significant improvement compared with our department’s historical data for neoadjuvant gastric cancer treatment. In the historical cohort, the pCR rate was 21.6%, 66.9% of patients achieved PR, the DCR was 94.5%, and the ORR was 71.5% — all of which were substantially lower than the results observed in the current OVs combined SOXP regimen (22).

Another key innovation of our study lies in the interventional arterial infusion of OVs directly targeting gastric cancer lesions. Notably, the efficacy and safety of H101 administered via this interventional approach for gastric cancer have not been previously reported. In our cohort, 25 patients tolerated H101 well, with no significant adverse events (AEs) observed. Fever, when present, was mild, no thrombocytopenia occurred, and no Grade 4 AEs were documented throughout the two cycles of neoadjuvant therapy. These findings confirm that the combination of H101 with the SOXP regimen effectively controls gastric cancer progression and may improve survival outcomes without increasing the incidence of complications.

The observed increase in circulating CD8^+^ T-cell counts following H101 administration is an encouraging finding that warrants careful interpretation. Consistent with published evidence demonstrating H101’s immunomodulatory properties (15, 16), we hypothesize that H101 may contribute to remodeling the gastric cancer TME by promoting the infiltration and/or expansion of cytotoxic T lymphocytes. This would be consistent with the proposed mechanism by which OVs enhance antitumor immunity by releasing TAAs, inducing immunogenic cell death, and activating innate and adaptive immune pathways (15, 16, 26, 27). However, it is important to emphasize that the current evidence is limited to peripheral CD8^+^ T-cell quantification, which reflects systemic rather than intratumoral immune changes. A definitive conclusion that the TME was converted from a “cold” to a “hot” phenotype cannot be drawn from these data alone, as comprehensive characterization of the tumor immune contexture would require additional analyses, including intratumoral immune cell profiling (e.g., CD8^+^, CD4^+^, FoxP3^+^ Tregs, tumor-associated macrophages), activation and exhaustion marker assessment (e.g., PD-1, TIM-3, LAG-3), cytokine profiling, and evaluation of antigen presentation pathways. Therefore, the proposed “cold-to-hot” conversion should be regarded as a plausible hypothesis consistent with the mechanistic literature, rather than a demonstrated biological effect, and must be tested rigorously in future prospective studies with comprehensive TME profiling.

The TME is enriched with immunosuppressive cells, and OVs can specifically modulate the proportion and functional properties of various immune cell subsets, thereby potentially reversing this immunosuppressive state (26). Regarding tumor-associated macrophages (TAMs), OVs may mediate the activation of relevant signaling pathways via released signaling molecules, promoting polarization from the pro-tumor immunosuppressive M2 phenotype toward the M1 phenotype—characterized by tumor cell phagocytosis, pro-inflammatory cytokine secretion, and T-cell activation (27, 28). Concurrently, OVs may reduce the proportion and suppressive capacity of regulatory T cells (Tregs) and decrease the number of myeloid-derived suppressor cells (MDSCs), thereby potentially alleviating the inhibitory effects of these cell populations on effector T cells (29). Furthermore, OVs can directly or indirectly enhance the cytotoxic activity of natural killer (NK) cells and may facilitate the intratumoral infiltration of effector CD8^+^ T cells and CD4^+^ helper T cells (17, 27). These mechanistic hypotheses, though supported by preclinical and early clinical evidence in other tumor types, remain to be directly validated in the gastric cancer setting.

OVs can be administered via multiple routes, including systemic intravenous injection, local injection into malignant serous effusions (e.g., pleural and ascitic fluids), and interventional arterial infusion for liver cancer (30). Systemic intravenous administration was excluded from our study due to two key limitations: hepatic sequestration of OVs significantly reduces the viral load reaching the tumor site, and the host’s innate immune response and antibody production further restrict viral proliferation and systemic spread. In our prior publications, we reported cases of colon cancer where endoscopic intratumoral injection of OVs achieved tumor downstaging (31). However, this local injection approach has inherent drawbacks for gastric cancer: the dense stroma and increased intratumoral pressure hinder deep penetration and homogeneous distribution of OVs. For instance, in diffuse lesions, viral concentration is high at the injection site but cannot be ensured in deep tumor regions distal to the injection point. Additionally, most gastric cancer patients present with ulcerative lesions, which inherently carry a high bleeding risk—improper local injection may exacerbate this risk. Endoscopic intratumoral injection also demands exceptional operational proficiency from endoscopists to avoid complications.

In contrast, interventional arterial perfusion offers distinct advantages, including high and homogeneous intratumoral drug concentrations. Notably, interventional therapy is recommended in clinical guidelines for the management of bleeding gastric cancer lesions (32), and our center has accumulated extensive experience with interventional perfusion for gastric cancer, which has confirmed its safety and feasibility (22). Therefore, we selected interventional arterial perfusion as the administration route for OVs, leveraging its dual benefits: achieving high local viral concentrations to maximize antitumor efficacy and enabling simultaneous hemostasis or bleeding prevention via interventional embolization, which addresses the bleeding risk associated with ulcerative gastric cancer lesions.

We also analyzed CTCs to evaluate the treatment impact on tumor metastatic potential. Mesenchymal CTCs are indicative of strong tumor metastatic and invasive capacity, while hybrid CTCs reflect an epithelial-mesenchymal transition (EMT) state associated with heightened metastatic potential. The numbers of both mesenchymal and hybrid CTCs decreased significantly post-treatment. These results not only validate the antitumor efficacy of H101 combined with the SOXP regimen but also suggest a potential reduction in the risk of distant metastasis, as reflected by the suppression of EMT-related and mesenchymal CTC subsets. These observations are preliminary and hypothesis-generating; prospective validation is required.

In conclusion, H101 combined with the SOXP regimen may represent a potential therapeutic option for patients with LAGC, and prospective studies with comprehensive assessment of both efficacy and toxicity will be needed in the future.

### 4.1 Limitations

This retrospective study has several limitations. First, retrospective studies are prone to selection bias, and the sample size is small. The limited number of relevant studies has led to uncertainties among patients regarding treatment efficacy and safety, resulting in a small number of enrolled cases. Prospective, large-sample clinical trials are required to obtain more convincing results. Second, this study only evaluated gastric cancer without distant metastasis; the efficacy of this regimen in stage IV gastric cancer was not assessed and requires dedicated future investigation. Third, there are no established references regarding the dosage of H101 for interventional therapy of gastric cancer; only the dosages used in interventional therapy for liver cancer and intratumoral injection for gastric cancer were taken as references, and insufficient dosage may exist for this approach. Fourth, as noted in the Discussion, the available data are insufficient to conclude that a definitive cold−to−hot TME conversion occurred; future studies should incorporate comprehensive intratumoral immune profiling.

## 5 Conclusions

This retrospective study indicates that the combination of H101 with the SOXP regimen exhibits promising short-term efficacy in the neoadjuvant treatment of LAGC, characterized by high ORR, DCR, R0 resection rate, and pCR rate. The preliminary evaluation shows that H101 administered via interventional therapy is safe in the treatment of LAGC. Additionally, the treatment-related toxicities are well-tolerated, supporting the potential clinical value of this therapeutic strategy for LAGC patients.

## Data Availability

The original contributions presented in the study are included in the article/**Supplementary Material**. Further inquiries can be directed to the corresponding author.

## Supplementary material

Supplementary Table 1 The tumor regression and type of PD-1 inhibitor Tumor regression (Y) group contains the patients from TRG 2,3 group and the tumor regression (N) group contains patients in the TRG 0,1 group.
