# Supplementary material for: Short-term efficacy and safety of recombinant human adenovirus type 5 combined with PD-1 immune checkpoint inhibitors and SOX regimen in neoadjuvant therapy of locally advanced gastric cancer: a retrospective study
Source: Front Oncol. 2026 Jul 15;16:1851169. doi: 10.3389/fonc.2026.1851169 (PMC13414111; doi:10.3389/fonc.2026.1851169)
Supplement: Supplementary Table 1 — The tumor regression and type of PD-1 inhibitor Tumor regression (Y) group contains the patients from TRG 2,3 group and the tumor regression (N) group contains patients in the TRG 0,1 group. [file DataSheet1.docx]

Figure S1 The tumor regression and type of PD-1 inhibitor

|  | Sintilimab | Tislelizumab | *P* value |
| --- | --- | --- | --- |
| Tumor regression(Y/N) |  |  | 0.87 |
| Y | 13 | 11 |  |
| N | 1 | 0 |  |

Tumor regression (Y) group contains the patients from TRG 2,3 group and the tumor regression (N) group contains patients in the TRG 0,1 group.
